# Knowledge Distillation in Video-Based Human Action Recognition: An Intuitive Approach to Efficient and Flexible Model Training

**DOI:** 10.3390/jimaging10040085

**Published:** 2024-03-30

**Authors:** Fernando Camarena, Miguel Gonzalez-Mendoza, Leonardo Chang

**Affiliations:** 1 School of Engineering and Science, Tecnologico de Monterrey, Nuevo León 64700, Mexico; 2 KODS.ai, Mexico City 11510, Mexico; leo@kods.ai

**Keywords:** video-based human action recognition, knowledge transfer, knowledge distillation, self-supervised action recognition

## Abstract

Training a model to recognize human actions in videos is computationally intensive. While modern strategies employ transfer learning methods to make the process more efficient, they still face challenges regarding flexibility and efficiency. Existing solutions are limited in functionality and rely heavily on pretrained architectures, which can restrict their applicability to diverse scenarios. Our work explores knowledge distillation (KD) for enhancing the training of self-supervised video models in three aspects: improving classification accuracy, accelerating model convergence, and increasing model flexibility under regular and limited-data scenarios. We tested our method on the UCF101 dataset using differently balanced proportions: 100%, 50%, 25%, and 2%. We found that using knowledge distillation to guide the model’s training outperforms traditional training without affecting the classification accuracy and while reducing the convergence rate of model training in standard settings and a data-scarce environment. Additionally, knowledge distillation enables cross-architecture flexibility, allowing model customization for various applications: from resource-limited to high-performance scenarios.

## 1. Introduction

Video-based human action recognition aims to understand a subject’s behavior, enabling core applications like video surveillance [1,2], content moderation [3], patient monitoring [4], and interactive gaming experiences [5].

Recognizing human actions poses significant challenges for computers even though it comes naturally to us humans [6]. State-of-the-art methods rely heavily on deep-learning approaches, meaning training a model requires significant computational resources [6]. As a result, developing more efficient training approaches for video-based human action recognition is crucial.

Transferring knowledge from one model to another is a common technique to reduce computational resource requirements, with transfer learning [7] and fine-tuning [8] being the most common techniques.

On the one hand, transfer learning [7] involves using a pre-existing model’s architecture and weights as a starting point to train a new model. On the other hand, fine-tuning [8] adds trainable layers to an existing pretrained model.

While transfer learning [7] and fine-tuning [8] offer improvements like reducing computational resource requirements, they have some limitations in their flexibility and efficiency. Since they rely on architectural cues of pretrained models, this makes the training process task-specific and limits the extracted knowledge of a model [6,9].

Knowledge distillation [10,11,12] is a widely used technique for creating a smaller version of a pretrained model that meets specific application needs, and it has recently been explored as a knowledge transfer technique. Current research primarily focuses on transferring knowledge in the domain of language models [13,14] and image classification tasks [10,15,16,17]. Nevertheless, as far as we know, the potential of knowledge distillation as a means of knowledge transfer, rather than just compression, has not been thoroughly investigated in video-based human action recognition, which is where our contributions mainly lie.

As a result, our work explores knowledge distillation as a knowledge transfer technique for boosting the training of self-supervised video models in three aspects: improving classification accuracy, accelerating model convergence, and increasing model flexibility under regular and limited-data scenarios.

We tested our method on the UCF101 dataset using differently balanced proportions: 100%, 50%, 25%, and 2%. Our findings suggest that using knowledge distillation as a guide for model training is more effective than traditional training methods while reducing the convergence rate of model training in standard settings and a data-scarce environment. Additionally, knowledge distillation offers cross-architecture flexibility, enabling model customization for different applications: from resource-limited to high-performance scenarios.

The rest of the document is organized as follows: Section 2 establishes the theoretical foundation. Section 3 details our experimental methodology. In Section 4, we analyze and discuss our findings, focusing on performance implications, efficiency, and scenarios with limited data. Lastly, Section 5 summarizes our findings, presents our conclusions, and suggests potential avenues for future research.

## 2. Related Work

This section has two main objectives. Firstly, in Section 2.1, we aim to clarify the concept of human action. Secondly, in Section 2.2, we provide an overview of human action recognition.

### 2.1. Breaking down the Concept of Human Action

A human action is a pattern formed by a sequence of gestures that both people and artificial sensors can recognize; let us imagine a scenario wherein one person greets another to explain the intuition of a sequence of gestures.

**Figure 1:**
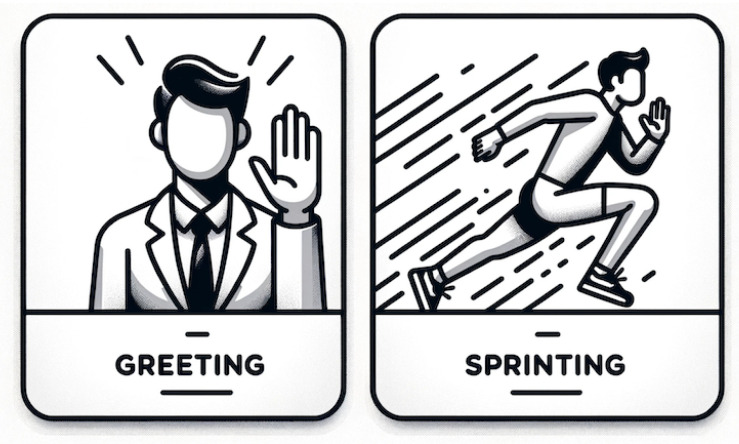
Human actions: When we think of greeting someone, we often picture a hand wave. On the other hand, when we imagine someone running, we visualize a more dynamic scene with the main movement happening in their legs.

A safe guess is to picture the familiar hand-waving as the representation of greeting, as Figure 1 suggests. Likewise, when we think of someone running, our attention naturally goes to the movement of his/her legs. Our brains have developed to link specific meanings or messages with a particular physical action that is commonly called “a human action” [18,19].

### 2.2. Overview of Human Action Recognition

This section provides the necessary background and context for our research and is divided into four parts. First, we introduce the field of human action recognition and its common challenges. Second, we provide an overview of the approaches taken in the field. Third, we review the current research in this area. Finally, we explain how our work fits the existing literature.

#### 2.2.1. Video-Based Human Action Recognition Field

Video-based human action recognition is an active field of research with ongoing developments. Its goal is to develop a model that can extract and understand the encoded message of human action, as Section 2.1 introduced.

Despite our natural talent to understand human actions, a computer faces different challenges. These can be divided into five areas [20]: action–class variability, sensor capabilities, environmental conditions, dataset restrictions, and computational constraints.

When discussing action–class variability, we have two types: intra-class variations, which refer to differences within specific action classes, and inter-class variations, which refer to the differences between various action classes [6]. In order to improve the accuracy of computer vision applications, it is crucial for models to address inter- and intra-class variations effectively.

On the other hand, despite being the most commonly used sensor for video action recognition, RGB cameras present challenges such as a restricted field of view and limited perspective, making it difficult for them to detect human actions accurately. Moreover, environmental conditions and the quality of the sensor’s images can significantly affect the model’s classification performance [6,19].

A significant challenge to constructing a high-classification model is the amount and quality of data used. There are two main approaches; creating datasets from scratch can ensure fitting the application’s specifications, but this can be resource intensive [21], and extracting data for some application domains can be difficult due to factors related to the nature of data, data privacy, or ethical considerations [22]. On the other hand, utilizing existing datasets may not adequately represent all the variations of target actions or fulfill the data dimensionality requirements [6]. Additionally, the degradation of publicly available datasets over time is a concern [6].

Finally, providing adequate computational resources is challenging when constructing video models for human action recognition [23]. On the one hand, most approaches use a supervised approach, which demands dealing with high-dimensional data and complex architectures [10,11,24]. On the other hand, specific applications require a fast inference response [9], and the model’s complexity may surpass the hardware’s processing capabilities [6,9].

#### 2.2.2. Approach Evolution

Early approaches for human action recognition were based on handcrafted methodologies [19,25], which are known for their manual feature engineering.

Nevertheless, owing to their performance and ability to extract video features without human engineering, deep learning approaches have set a novel standard for human action recognition [1,26,27].

However, applying deep learning methods to action recognition was not straightforward. Early approaches based on traditional CNNs do not outperform handcrafted methods since human actions are defined into spatial–temporal features and traditional neural networks. Therefore, exploring how to model temporal information became the research focus, and researchers arrived at a two-stream network with two separate networks to process the spatial and temporal information separately. The next step in video-based action recognition was the two-stream inflated 3D ConvNet (I3D) [2] architecture. I3D [2] demonstrates that 3D convolutional networks can be pretrained. From this point, multiple video architectures emerged, including R3D [28] and R(2+1)D [29].

Transferring knowledge from one model to another is a common way to reduce computational resource requirements [7]. Two commonly used techniques in the literature are transfer learning and fine-tuning [7,8].

Transfer learning [7] involves using the architecture and weights of a preexisting model to train a new model, which is particularly effective when the new task is similar to the original task on which the preexisting model was trained. On the other hand, adding trainable layers to an existing pretrained model and training only these layers on a new task is called fine-tuning [8].

While transfer learning [7] and fine-tuning [8] can be beneficial for reducing computational resource requirements and speeding up the training process, they have some limitations. Due to their reliance on pretrained models’ architectural cues, these techniques can significantly limit the knowledge extracted from the model, making the training process highly specific to a particular task [6,9].

#### 2.2.3. Current Research

Current research in video-based learning can be divided into six directions: new architectures, novel learning paradigms, pretraining and knowledge transfer, exploring video modalities, and cross and multimodal learning.

Due to the growing popularity of transformers in natural language processing, their application to human action recognition has emerged [30]. Conversely, deep learning methods rely extensively on labeled datasets; therefore, there is a need for a more efficient and less resource-intensive learning paradigm [10,24,31,32,33]. Some of the novel learning paradigms include semi-supervised learning [31], weakly supervised learning [32], and self-supervised learning (SSL) [24,33].

Self-supervised learning leverages unlabeled data by generating a supervision signal without manual annotation, as inspired by our natural learning processes [33]; one promising approach in image-based tasks is few-shot learning. It allows for learning with limited data, reduces computational demands, and generalizes to new action classes [21,34,35,36,37].

Transfer learning [7] and fine-tuning [8] have been demonstrated to be beneficial for improving the performance and convergence of a model. Novel approaches have emerged, including knowledge distillation (KD) [10,11,12]. KD is a widely used technique for creating a smaller version of a pretrained model that meets specific application needs. However, recently, it has been explored for its potential as a knowledge transfer technique for image tasks [10]. However, applying knowledge distillation for knowledge transfer for video-based human action recognition remains unexplored.

Another significant factor is related to video modalities; most works use the RGB modality, but the application of other modalities could improve the features extracted in specific scenarios [30]. In general, video modalities can be divided into visual and non-visual modalities [30]. Potential visual modalities includes RGB [38], Skelethon [39,40,41,42], depth [43], infrared [44], and thermal [45]. On the other hand, emerging nonvisual modalities include audio [46], acceleration [47], radar [48], and WiFi [49].

Our interaction with the world is multimodal; therefore, developing models that can leverage the strength of each modality may improve performance, robustness, and privacy. Two common ways to use different modalities are multi-modal [33,50] and cross-modal [51].

#### 2.2.4. How Our Work Fits in the Literature

We set five current research paths: new architectures, novel learning paradigms, pretraining and knowledge transfer, exploring video modalities, and cross and multimodal learning. Our work fits in with the knowledge transfer research path since our primary focus is to explore novel knowledge transfer methods that do not depend on architectural cues, which is helpful for ensuring the transferability of knowledge for emerging novel architectures. Additionally, our work is done in a self-supervised environment and focuses on testing in low-data settings, which is also considered a current research path. Further, we believe that a potential future direction of our work will be in cross-modality learning scenarios, which is challenging because of the disjunctive feature space of the modalities.

Regarding similar works, current research in knowledge distillation has primarily focused on transferring knowledge in the domain of language models [13,14] and image classification tasks [10,15,16,17]. Yet there have been fewer works in other fields, such as object detection [52] and segmentation [53], domain generalization [54], and video classification. Our work contributes to exploring knowledge distillation in the video-based action recognition field [55].

Knowledge distillation has been adopted for language models as a response to the trend of building larger pretrained models efficiently. Qin et al. [13] propose a knowledge inheritance (KI) framework that combines self-learning and teacher-guided learning to train large-scale language models using previously pretrained models. Its core idea relies on the inclusion of auxiliary supervision with a dynamically balancing weight to reduce the influence of the teacher model in the late stage of the training. Similarly, Chen et al. [14] propose bert2BERT: a pretrained framework with the core idea of using smaller teachers to create a larger student model.

Knowledge distillation has also been explored for image classification tasks. Xu et al. [10] present SSKD, which combines self-supervision and knowledge distillation to enable a model-agnostic approach that outperforms the state-of-the-art models on the CIFAR100 dataset. Park et al. [15] aim to understand what makes a teacher model friendly to a student to increase classification performance. Rajasegaran et al. [16] explore a two-stage learning process to extract better model representations that enable good performance for few-shot learning tasks. Yang et al. [56] explore using hierarchical self-supervised knowledge distillation that adds auxiliary classifiers to intermediate feature maps with the goal of generating diverse self-supervised knowledge that can be transferred to the student model. Xu et al. [17] suggest collaborative knowledge distillation between the teacher model and a self-distillation process. Wen et al. [57] introduce the concepts of knowledge adjustments and dynamic temperature distillation to penalize inadequate supervision and, therefore, improve student learning. Finally, self-supervised teaching assistants (SSTA) [58] focus on improving visual transformers using two teacher heads, either supervised or self-supervised, along with a selection method to mimic the attention distribution.

Further research is required in other domains, but the success of knowledge distillation in language and image classification tasks shows potential usefulness in other fields. The MobileVos framework [53] aims to achieve real-time object segmentation on resource-constrained devices by combining KD and contrastive learning. Zhang et al. [52] focus on object detection using KD to address two fundamental problems: the imbalance between foreground and background pixels and the lack of consideration of the pixel’s relations. Domain generalization is explored by Huang et al. [54], where the student is encouraged to learn image representations using the teacher’s learned text representations. Finally, Dadashzadeh et al. [55] introduced auxSDX, which adds an auxiliary distillation pretraining phase for video representations. Our work is fundamentally different from auxSDX [55] since its core contributions rely on the introduction of a novel self-supervised pretext task that uses the distilled knowledge from the teacher. In contrast, despite also working on a self-supervised methodology, we explore a more general and flexible way to include the guidance of the teacher model by focusing on using the logits to understand how the probability distributions differ between the models. Another difference is that in Dadashzadeh et al.’s [55] work, the teacher and student models share the same architectural settings, which differs from our flexibility goal.

## 3. Methodology

Our proposal consists of two components: self-supervised learning (SSL) and knowledge distillation (KD), which are discussed in Section 3.1.

Our work explores the implications of KD as a training guide for a self-supervised action recognition model, which has not been fully explored in video settings, as shown in Section 3.4.

### 3.1. Preliminaries

In this section, we explore two key works that serve as the foundation of our work. In Section 3.2, we explore self-supervised learning, and in Section we explore knowledge distillation as a transfer mechanism.

### 3.2. Self-Supervised Video-Based Model Training

The goal of self-supervised learning (SSL) [33] is to extract representative feature representations from videos without a manually annotated dataset. SSL [33] has two main approaches: pretext tasks and contrastive learning.

#### 3.2.1. Pretext Tasks

Pretext tasks [59] define classification tasks that learn low-level features that could be refined to the target task. Determining the optimal classification task is still an unresolved problem. However, our study investigates a pretext task that relies on video transformation.

The objective is straightforward: the network’s task is to determine the transformation applied to the input video clip. As a result, the network gains valuable insights from the video without requiring explicit training on labeled data.

Formally, let us define $f_{\theta }\left(\cdot \right)$ as the backbone network to extract spatio–temporal features, $t\left(\cdot \right)$ as the transformation function, $v_{i}$ as video *i*, $x_{v_{i}}$ as the video clip for video *i*, and *y* as the transformation label.

The first step is to apply the *y* transformation to video clips $x_{v_{i}}$ using the transformation function $t\left(x_{v_{i}},y\right)$. Our study explores five types of transformations: unchanged, rotation, spatial permutation, temporal adjacent shuffling, and temporal shuffling. The rotation transformation randomly rotates a video clip between 90 and 270 degrees. Spatial permutation rearranges the quadrants of a video clip. The video clip is initially divided into four equal quadrants and is then randomly shuffled. Temporal adjacent shuffling randomly reorganizes segments of a video by dividing it into four temporal sections and swapping two adjacent sections. Finally, temporal shuffling divides the video clip into four temporal segments and replaces the second segment with the fourth segment.

The backbone network $f_{\theta }\left(t\left(x_{v_{i}},y\right)\right)$ uses the output of the transformation function to extract visual and temporal features.

As a usual practice in classification problems, we use cross entropy as the loss function, as shown in Equation (1).

(1) $$L_{crossentropy}\left(f_{\theta }\left(t\left(x_{v_{i}},y\right)\right),y\right)$$

#### 3.2.2. Contrastive Learning

Contrastive learning emphasizes differences between video clips by comparing their similarities in a shared space [24,59].

Let us consider $x_{v_{i}}$ and $x_{v_{j}}$ as video clips from videos *i* and *j*. Then, $x_{v_{i}}^{1}$$x_{v_{j}}^{1}$ and $x_{v_{i}}^{2}$$x_{v_{j}}^{2}$ represent the second clip from videos *i* and *j*, respectively.

The network is fed with a pair of video clips, and the goal is to determine if the clips are from the same distribution. For example, while clips $x_{v_{i}}^{1}$ and $x_{v_{i}}^{2}$ are considered from the same distribution, clips $x_{v_{i}}^{1}$ and $x_{v_{j}}^{2}$ are treated as distinct.

To project the features onto a shared space, we require a projector network denoted as $h\left(\cdot \right)$, where a common approach is to use a two-linear-layer multi-layer perceptron (MLP), as explained by Tao et al. [59].

Therefore, the features projected from $x_{v_{i}}$ are $h\left(f\left(x_{v_{i}}\right)\right)$, which is simplified to $z_{v_{i}}$. The similarity distance is computed using a dot product, which is represented as $D\left(z_{v_{i}},z_{v_{j}}\right)$.

The goal is to minimize Equation (2), which is composed of two parts that are defined in Equations (3) and (4).

(2) $$\mathrm{minimize}L^{v_{i}}=L^{v_{i}^{1}}+L^{v_{i}^{2}}$$

where $L^{v_{i}^{1}}$[59] aims to compute the similarity between the feature vectors $v_{1}^{1}$ and $v_{i}^{2}$ using the dot product as the *D* distance function, as defined in (3):

(3) $$L^{v_{i}^{1}}=-\log\frac{D\left(z_{v_{i}}^{1},z_{v_{i}}^{2}\right)}{D\left(z_{v_{i}}^{1},z_{v_{i}}^{2}\right)+\sum_{j\neq i}D\left(z_{v_{i}}^{1},z_{v_{j}}^{1}\right)}$$

$L^{v_{i}^{2}}$ is defined in Equation (4) and aims to compute the similarity between the feature vectors $v_{i}^{2}$ and the negative sample.

(4) $$L^{v_{i}^{2}}=-\log\frac{D\left(z_{v_{i}}^{1},z_{v_{i}}^{2}\right)}{D\left(z_{v_{i}}^{1},z_{v_{i}}^{2}\right)+\sum_{j\neq i}D\left(z_{v_{i}}^{2},z_{v_{j}}^{2}\right)}$$

#### 3.2.3. Merging Pretext and Contrastive Learning

Pretext tasks and contrastive learning provide unique insights into understanding visual data [59]. Pretext tasks emphasize a sample’s innate details, allowing one to understand the intra-class variations. In contrast, contrastive learning focuses on identifying the differences between one instance and another, which helps with understanding of inter-class differences.

**Figure 2:**
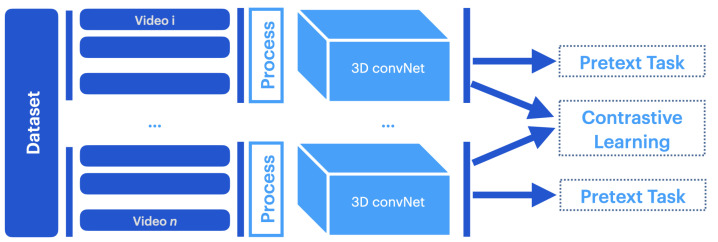
The pretext–contrastive learning (PCL) [59] framework. PCL is a joint framework that combines the pretext task and contrastive learning methods. Adapted from [59].

Pretext–contrastive learning (PCL) combines pretext tasks and contrastive losses to ensure the network benefits from a local and global understanding of the data, as shown in Figure 2.

Merging both approaches is done by the linear combination shown in Equation (5), where $L_{\mathrm{pretext}\;}$ and $L_{\mathrm{contrast}\;}$ are computed using Equations (3) and (4), respectively, and weight α is used to balance the losses between the pretext tasks and contrastive learning [59].

(5) $$L_{\mathrm{total}\;}=L_{\mathrm{pretext}\;}+\alpha L_{\mathrm{contrastive}\;}$$

### 3.3. Knowledge Transfer by Knowledge Distillation

**Figure 3:**
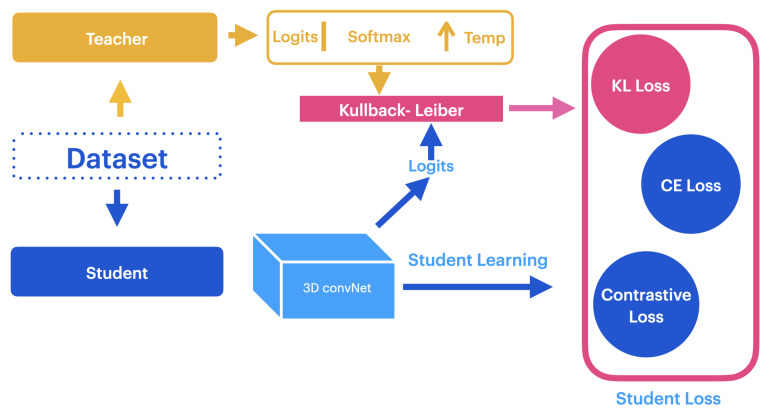
Representation of the teacher–student knowledge distillation (KD) framework. The main process involves computing the Kullback–Leibler (KL) divergence between the softened output probabilities of both models.

A common method to decrease computational resource requirements and reduce dependence on labeled data is transferring knowledge between models [10,12,24]. This work implements a teacher–student knowledge distillation framework to transfer knowledge between models with different architectures, as shown in Figure 3.

The teacher–student approach encourages students to redefine their learning direction based on the teacher model’s knowledge direction. Formally, the student and pretrained teacher model are defined as $f_{\theta }\left(\cdot \right)$, with no restrictions on architectures. Both networks are expected to have a classifier head $p\left(\cdot \right)$ that maps the feature vector to the action class probabilities.

The relationship between the student and teacher models is established through the Kullback–Leibler (KL) divergence [12]. This measure assesses the dissimilarity between two probability distributions, enabling the student model to measure how much they differ and to adjust their weights to minimize the gap, progressively gaining expertise. A temperature τ must soften the output probabilities used to compare the probability distributions. Softening increases the differences between action classes, especially in cases where the teacher model’s output values are close to 0 or 1 [10,12]. As in [10,12], we set the τ value to four using the log softmax functions available in PyTorch.

KL Divergence is used to calculate the knowledge distillation loss, as shown in Equation (6)

(6) $$L_{kd}=-\tau^{2}\sum_{x∼D}\sum_{i=1}^{C}p_{t}^{i}\left(x;\tau \right)\log\left(p_{s}^{i}\left(x;\tau \right)\right)$$

The knowledge distillation loss function $L_{kd}$ is determined by summing the product of the teacher network’s probabilities $p_{t}^{i}\left(x;\tau \right)$ and the logarithm of the student network’s probabilities $p_{s}^{i}\left(x;\tau \right)$ for every video *x*.

Our goal is to not only create a smaller teacher model but also to improve its performance and enable continued student training. Thus, the complete loss function, shown in Equation (7), is a linear combination of $L_{kd}$ and $L_{student}$, where $L_{student}$ is cross entropy and λ is a balancing weight.

(7) $$L_{student}=L_{student}+\lambda L_{kd}S$$

### 3.4. Experimental Design

This section provides an overview of the experimental design of the study. Section 3.4.1 outlines the research objectives, and Section 3.4.2 details the experimental setup.

#### 3.4.1. Research Objectives

Our primary objective is to investigate the effectiveness and efficiency of knowledge distillation (KD) for training a video-based model for human action recognition. Therefore, we structure our experiments into three main areas:

- Performance implications: We studied how knowledge distillation affects the model’s performance. We hypothesized that the student model could benefit from the teacher model’s experience, leading to better accuracy and recognition rates. Our question: Is training the model with knowledge distillation better than training it from scratch for classification accuracy?
- Convergence rate efficiency: We aim to assess if KD can speed up the convergence, which would reduce the training time and resources required. We are interested in the evolution of performance during the early, middle, and late training stages to understand how the model progresses by comparing the rate of convergence and epochs needed to reach accuracy milestones. Our objective is to determine how KD affects the rate of convergence of model training.
- KD in data-limited situations: Training in low-data environments is challenging. We aim to understand if KD can leverage distilled knowledge from a teacher model to provide an advantage in such scenarios. To assess performance in low-data regimes, we conducted experiments using differently balanced proportions of the dataset. We check how reducing the data affects model performance and compare these results to the performance of a model trained from scratch.

#### 3.4.2. Experiment Setup

Our workstation has an Intel^®^ Xeon(R) Silver 4210R CPU and an NVIDIA A6000 GPU. This GPU handles deep learning workloads and ensures faster training and efficient parallel processing. We standardized our software development process using Docker image *nvcr.io/nvidia/pytorch:21.04-py3* from NVIDIA’s NGC catalog, which has all the necessary dependencies optimized for GPU acceleration.

Additionally, we configured the PyTorch backend cuDNN to run in a deterministic mode with a fixed seed value of 0, reducing the neural networks’ randomness and reinforcing our computational processes’ reproducibility.

### 3.5. Training Setup

We use three main architectures as backbone networks trained using the hyperparameters shown in Table 1; for data preprocessing and sequence generation, we used a video clip length of 10 frames, an interval of 8, and a tuple length of 3, as the PCL [59] approach suggests.

We set the learning rate to $1\times 10^{-2}$ and the momentum to $9\times 10^{-1}$ to ensure stability and faster convergence of our model. We applied weight decay of $5\times 10^{-4}$ to prevent overfitting. For parallel data loading, we employed 16 workers with a mini-batch size of 16. We used data augmentation techniques to improve the model’s generalization, including resizing the images to dimensions of 128 × 171 and then randomly cropping them to a 112 × 112 size. We also applied random color jittering with a probability of 0.8, which adjusted brightness, contrast, and saturation by 40% and hue by 10%. Grayscale augmentations with a 20% probability and Gaussian blur with a 50% probability were also used, with a variable kernel size ranging from 0.1 to 2. We trained the model for 200 epochs to ensure thorough exploration of its capabilities.

#### 3.5.1. Model Architectures

We experimented with three video architectures—namely, C3D [10], R3D [28], and R(2+1)D [29]—to serve as backbones for the PCL [59] framework. Testing different architectures can reduce bias and increase our understanding of knowledge transfer effectiveness. Combining architectures with varying complexities helps us to understand the capabilities in different hardware scenarios, including networks of different sizes.

R3D [28] and R(2+1)D [29] are deep learning models for recognizing human actions in videos that were inspired by the well-known ResNet architecture, which employs spatial and temporal residual layers to analyze the spatial and temporal aspects of a video.

The C3D [10] architecture is a 3D convolutional neural network composed of eight blocks of 3D convolutional layers.

#### 3.5.2. The UCF101 and HMDB51 Datasets

**Figure 4:**
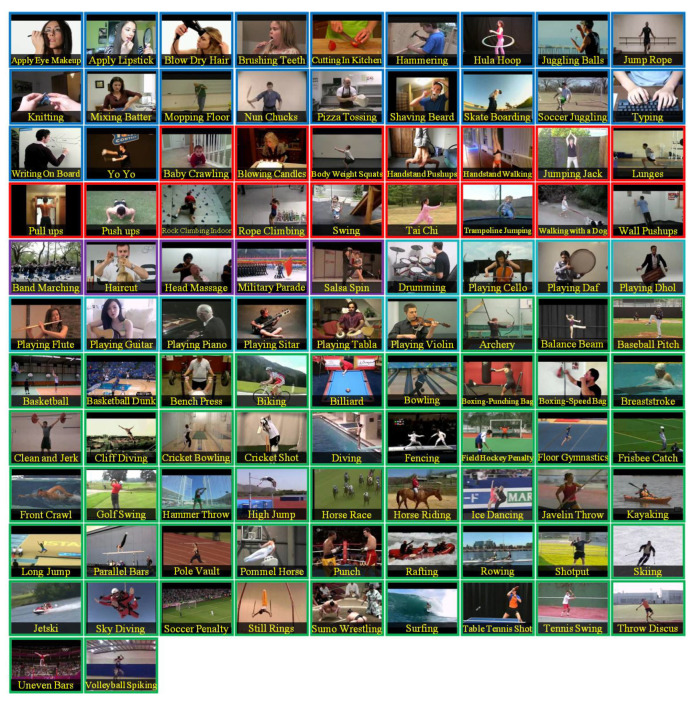
Overview of the 101 distinct human action categories as presented in the UCF101 dataset [60]. Adapted from [60].

The UCF101 [60] dataset offers a wide range of video sequences that serve as the foundation for assessing the effectiveness of human-action recognition approaches. It comprises 13,320 video clips extracted from YouTube and representing 101 types of human action, as shown in Figure 4. These actions cover a wide range of activities and are organized into different categories; human–object interactions refer to activities that require direct contact with objects. Body-motion-centric actions mainly involve specific bodily movements. Human–human interactions include activities that concern multiple individuals. Musical instrumental playing involves individuals playing instruments. Finally, sporting actions are related to athletic purposes. The dataset offers realistic sample diversities, including different resolutions, lengths, and quality levels.

**Figure 5:**
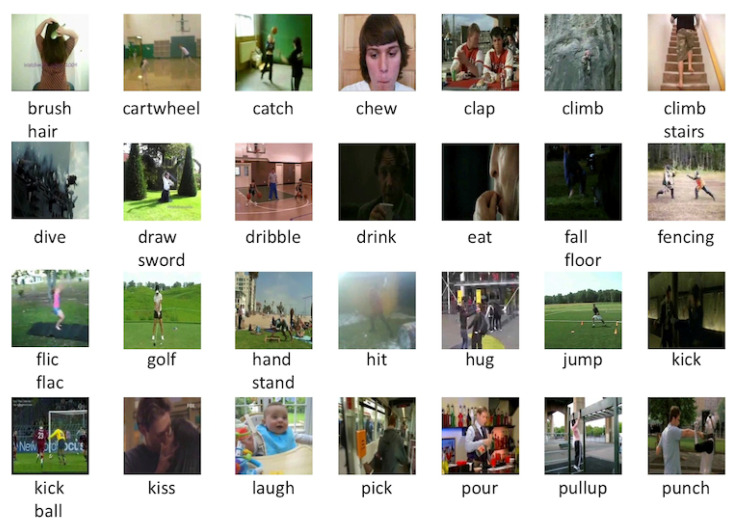
Overview of the 51 distinct human action categories presented in the HMDB51 dataset [61]. Adapted from [61].

The HMDB51 [61] dataset is an effective benchmark in video-based human action recognition. The dataset comprises 6849 video clips systematized into 51 action classes, as shown in Figure 5. These classes include facial actions and general body movements and cover human-to-human and human–object interactions. The dataset contains videos from various sources, resulting in diverse qualities, resolutions, and durations.

#### 3.5.3. Experiments

We compared knowledge distillation (KD) models to those trained from scratch in experiments, as summarized in Table 2.

For the R3D [28] student architecture, we trained two KD models: one with C3D [10] and the other using R(2+1)D as its teacher. Both models were trained on the full UCF101 dataset with a self-supervised methodology and fine-tuned on the HMDB51 dataset. We also trained an R3D [28] model from scratch.

We repeated this process for the C3D [10] student architecture, training two KD models guided by R3D [28] and R(2+1)D, respectively, and one model from scratch.

Lastly, we conducted experiments with R(2+1)D [29] as the student architecture. For the KD-trained models, we used C3D [10] and R3D [28] as teachers, and one model was trained from scratch.

We compared the model’s accuracy during different training stages to study whether knowledge distillation can speed up model training and save computational resources. The early (0–50 epochs), middle (50–150 epochs), and late (150–200 epochs) stages were analyzed using the same runs as presented in Table 2.

Finally, to evaluate model effectiveness with limited data, we repeated the experiments shown in Table 2 but used subsets of 50%, 25%, and 2% of the entire dataset.

We followed a specific methodology to create a subset representing X percent of the dataset. First, we randomly selected X percent from action class 1, then X percent from action class 2, and continued this process until we reached action class n. This method ensured that our subset was congruent with the complete set.

## 4. Results and Discussion

Section 4.1 assesses the impact of KD on the model’s performance during training, and Section 4.2 explores its impact on the convergence rate. Finally, we explore the resilience of the models in low-data scenarios in Section 4.3 and its performance in cross-architecture settings in Section 4.4.

### 4.1. Performance Implications

Table 2 shows the accuracy performance using the experimental settings described in Table 3. We presented the accuracy on the UCF101 [60] dataset and its fine-tuned value for the HMDB51 [61] dataset.

The best model for training an R3D [28] model was the KD configuration using the R(2+1)D teacher on the UCF101 and HMDB51 datasets. A similar finding is given while training a C3D [10] model, where both KD models outperform the scratch model, and the model with the R(2+1)D [29] teacher improved the performance by almost 8 percent. Finally, R(2+1)D [29], the student model, presents a dynamic similar to previous architectures in which both models guided by KD improve the accuracy.

Table 3 shows a pattern that methods boosted using KD tend to outperform training from scratch regardless of the architecture of their teacher models, suggesting that KD is an effective way to transfer knowledge from teacher to student models.

The performance increase on the HMDB51 dataset was slight since we employed a fine-tuning method on this dataset. Despite the low performance, we observe that KD-boosted models perform better.

### 4.2. Convergence Rate Efficiency

Our second goal is to understand our method’s convergence capabilities. While accuracy is essential, reducing the training time and computational resource requirements is crucial for some application domains.

To assess the model’s capabilities, we evaluated its accuracy performance on the test sets of the UCF101 dataset at three different training stages: early (first 50 epochs), mid (first 150 epochs), and late (usual training process). Our findings are presented in Table 4.

Table 4 compares the accuracy performance between a model’s evaluation and test sets at the early, middle, and late stages of training. We conducted this analysis on the C3D [10] model and found that its performance on the validation set was similar to that of the scratch one. However, when we compared the models on the test set, we observed a significant difference between them, indicating that using the KD-guided models results in better generalization of unseen data. Additionally, the performance of both C3D KD-guided models in the early stage outperformed by more than 7 percent of the model trained from scratch in the complete settings, suggesting that KD-guided models significantly reduce the computational resources required to train a model. During the middle stage of training, models show stability and a constant increase in their performance. During the late stage, all models display a similar dynamic, with KD-guided models achieving the best performance.

The R3D [28] architecture presents a dynamic similar to that of C3D. On the one hand, KD-guided models performed better than the model from scratch in almost every stage, and the one using R(2+1)D [29] as the teacher outperformed the fully trained scratch model in an early stage of training. In the middle and late stages, all models presented a stable increase, and in the late stage, the scratch model closed the gap with its KD counterpart.

Finally, the R(2+1)D [29] model behaves similarly to previous architectures. The KD models with C3D [10] and R3D [28] teachers achieved early stage performances of 43 and 48 in contrast to the scratch model’s 41, meaning the KD models outperformed the model trained from scratch in the early stages.

The consistency in the KD model’s superior early stage performance highlights the method’s robustness for boosting the generalization capabilities of the approach.

### 4.3. Robustness in Low-Data Scenarios

This section explores the robustness of models when trained in low-data scenarios using the settings from Section 3. Training deep learning models in low-data settings can be challenging due to the strong correlation between data dimensionality and model performance. In Section 4.3.1, Section 4.3.2 and Section 4.3.3, we review knowledge distillation performance using the R3D, R(2+1)D, and C3D architectures with 2, 25, and 50 percent of the original training set.

#### 4.3.1. Performance Analysis Using Two Percent of the Training Set

Table 5 shows the performance during the initial, middle, and late training of a C3D model under a two-percent subset of the dataset. Similar to its performance using the complete dataset, the KD-guided models showed better accuracy in the early stage compared to the scratch model, indicating that the feature representations learned from the teacher model improve the generalization of unseen data. In the middle and late stages of the training, all models showed signs of convergence and possible overfitting due to the low amount of data used.

Table 5 displays the performance of the C3D [10] model. In the early stage, the R(2+1)D-guided model performed similarly to the scratch model, while the C3D-guided model outperformed the scratch model. As the training progressed, both KD models outperformed the scratch model. Eventually, all models reached the limit of their classification accuracy, indicating that there is little room for improvement due to the limitations of the data used.

Finally, the performance of the R(2+1) shown in Table 5 confirms the behavior of the previous architectures. In the early stage of training, both KD models tend to outperform the scratch model; the performance of the scratch model slightly increases in the middle stage. Similarly, at the end of the training stage, the scratch model performs at the level of the KD-guided models, suggesting possible overfitting to the dataset.

#### 4.3.2. Performance Analysis Using 25 Percent of the Training Set

This section explores the implications of using a proportion of 25% of the training set, as shown in Table 6. For the C3D architecture, similar to our experiments using two percent, KD-guided models achieved better performance than the scratch model, especially the R(2+1)D-guided model, which showed a significant increase in classification accuracy. Training for a longer time provides a slight improvement to accuracy for all models, while knowledge distillation models. Training the models for the complete 200 epochs does not improve the classification performance, suggesting overfitting to the dataset.

In the case of the R3D model, the R(2+1)D-guided and the scratch models showed similar performance, while the C3D-guided model outperformed both models by a large margin. In the middle and late stages, all models achieved a plateau in classification accuracy.

For the last model, R(2+1)D [29], the KD-guided models benefit from the guidance and outperform the scratch model in the early stage of training; this is congruent with the performance of the other architectures and its performance using a minimal proportion of the dataset.

#### 4.3.3. Performance Analysis Using 50 Percent of the Training Set

This section examines the implications of applying the KD to 50% of the dataset. All tested architectures, shown in Table 7, are consistent with our previous experiments; the KD-guided model outperforms scratch models in the early stage of the training, and during the late stage of the training, KD-guided models overfit and the scratch model closes the performance gap, suggesting that KD-models better learn representative visual features in an early stage, reducing the computational resource requirements.

### 4.4. Cross-Architecture Comparison

We aim to determine the transferability of KD-guided models across different architectures. In Section 3 and Section 4.3, we explored the performance of KD-guided models in standard and low-data settings. The experimental designs, shown in Table 2, examine multiple settings using different teacher architectures to train the student model.

Our insights showed that most of the tested model configurations outperformed scratch models by having significant generalization capabilities that enabled the achievement of high classification performance in the early stage of the training and, as a consequence, reduced computational resource requirements. Therefore, KD transfers knowledge between various architectures, emphasizing the method’s flexibility and adaptability. Moreover, when challenged with limited-data scenarios, our KD approach consistently delivered robust improvement to the training process independent of the architecture used.

## 5. Conclusions and Future Work

Our main objective in this work was to study the implication of using KD-guided models to train self-supervised video-based human action recognition based on three main aspects: its performance, its convergence rate, and its robustness in low-data scenarios.

We conducted comprehensive experiments comparing KD-guided and scratch models and focusing on three distinct architectures: R3D, C3D, and R(2+1)D. Our key insights were that KD-guided models outperform the scratch model while increasing its generalization capabilities on unseen data. Additionally, models guided by KD achieved faster convergence rates, with a few epochs generating higher classification accuracy than a scratch model achieved in four times more epochs, thereby conserving computational resources.

Finally, we experimented with smaller proportions of the dataset to test KD’s ability to work with limited data. Despite facing some overfitting, the KD-guided models showed consistently higher generalization in the early stage of the training compared to the scratch model.

All experiments were conducted using a cross-architectural setting, which highlights the versatility of KD-guided methods as a knowledge transfer technique and enables the training of custom models that meet specific application constraints, whether the goal is to develop leaner models for resource-constrained devices or more powerful models.

There are several directions for future research, including exploring new datasets and application domains, exploring novel techniques for feature assessment, and developing novel methods to compare the knowledge of the models and their application to transfer other facilities of methods and modalities.

## Figures and Tables

**Table 1:** This table overviews the essential training parameters and their corresponding values for our video-based human action recognition experiments.

Parameter	Value
Architecture	R3D [28], C3D [10], R(2+1)D [29]
Video Clip Length (frames)	10
Interval	8
Tuple Length	3
Learning Rate	$1\times 10^{-2}$
Momentum	$9\times 10^{-1}$
Weight Decay	$5\times 10^{-4}$
Mini Batch Size	16
Workers	16
Modality	res
Weight Contrastive Loss	0.5
MLP Head	For Contrast
Augmentation	True
Epochs	200

**Table 2:** Experimental configurations: This table provides an overview of the experiments employed in our study. It details several vital aspects, including the chosen method (KD-guided or Scratch), the student architecture (e.g., R3D, C3D, or R(2+1)D), the dataset (UCF101), the presence of a teacher architecture (if applicable), and the dataset used for fine-tuning (HMDB51).

Method	Student	Dataset	Teacher	Fine-Tune
KD	R3D [28]	UCF101	C3D [10]	HMDB51
KD	R3D [28]	UCF101	R(2+1)D [29]	HMDB51
Scratch	R3D [28]	UCF101	None	HMDB51
KD	C3D [10]	UCF101	R3D [28]	HMDB51
KD	C3D [10]	UCF101	R(2+1)D [29]	HMDB51
Scratch	C3D [10]	UCF101	None	HMDB51
KD	R(2+1)D [29]	UCF101	C3D [10]	HMDB51
KD	R(2+1)D [29]	UCF101	R3D [28]	HMDB51
Scratch	R(2+1)D [29]	UCF101	None	HMDB51

**Table 3:** Performance implications based on classification accuracy of using knowledge distillation (KD) as a guide for training a model versus training it from scratch on the UCF101 and fine-tuned to the HMDB51.

Method	Dataset	Student	Teacher	UCF101	HMDB51
KD	UCF101	R3D	C3D	0.45	0.61
KD	UCF101	R3D	R(2+1)D	0.48	0.62
Scratch	UCF101	R3D	None	0.47	0.60
KD	UCF101	C3D	R3D	0.55	.61
KD	UCF101	C3D	R(2+1)D	0.52	0.62
Scratch	UCF101	C3D	None	0.46	0.60
KD	UCF101	R(2+1)D	C3D	0.46	0.62
KD	UCF101	R(2+1)D	R3D	0.48	0.60
Scratch	UCF101	R(2+1)D	None	0.44	0.62

**Table 4:** Convergence rate efficiency: this table shows the classification accuracy during different stages of training.

Method	Student	Dataset	Teacher	Early Stage	Mid Stage	Late Stage
KD	R3D	UCF101	C3D	0.44	0.45	0.45
KD	R3D	UCF101	R(2+1)D	0.48	0.48	0.48
Scratch	R3D	UCF101	None	0.44	0.47	0.47
KD	C3D	UCF101	R3D	0.51	0.55	0.55
KD	C3D	UCF101	R(2+1)D	0.52	0.52	0.52
Scratch	C3D	UCF101	None	0.44	0.46	0.46
KD	R(2+1)D	UCF101	C3D	0.43	0.46	0.46
KD	R(2+1)D	UCF101	R3D	0.48	0.48	0.48
Scratch	R(2+1)D	UCF101	None	0.41	0.44	0.44

**Table 5:** Convergence rate efficiency: this table shows the classification accuracy using 2 percent of the dataset during different stages of training.

Method	Student	Dataset	Teacher	Early Stage	Mid Stage	Late Stage
KD	R3D	UCF101	C3D	0.42	0.43	0.44
KD	R3D	UCF101	R(2+1)D	0.29	0.38	0.39
Scratch	R3D	UCF101	None	0.25	0.34	0.34
KD	C3D	UCF101	R3D	0.26	0.44	0.44
KD	C3D	UCF101	R(2+1)D	0.25	0.40	0.40
Scratch	C3D	UCF101	None	0.24	0.33	0.36
KD	R(2+1)D	UCF101	C3D	0.29	0.39	0.39
KD	R(2+1)D	UCF101	R3D	0.28	0.36	0.36
Scratch	R(2+1)D	UCF101	None	0.24	0.38	0.38

**Table 6:** Convergence rate efficiency: this table shows the accuracy performance using 25 percent of the dataset during different stages of training.

Method	Student	Dataset	Teacher	Early Stage	Mid Stage	Late Stage
KD	R3D	UCF101	C3D	0.46	0.46	0.46
KD	R3D	UCF101	R(2+1)D	0.43	0.45	0.47
Scratch	R3D	UCF101	None	0.41	0.45	0.45
KD	C3D	UCF101	R3D	0.50	0.54	0.54
KD	C3D	UCF101	R(2+1)D	0.53	0.56	0.56
Scratch	C3D	UCF101	None	0.48	0.49	0.51
KD	R(2+1)D	UCF101	C3D	0.42	0.45	0.46
KD	R(2+1)D	UCF101	R3D	0.45	0.46	0.46
Scratch	R(2+1)D	UCF101	None	0.40	0.45	0.47

**Table 7:** Convergence rate efficiency: this table shows the accuracy performance using 50 percent of the dataset during different stages of training.

Method	Student	Dataset	Teacher	Early Stage	Mid Stage	Late Stage
KD	R3D	UCF101	C3D	0.42	0.44	0.45
KD	R3D	UCF101	R(2+1)D	0.40	0.42	0.42
Scratch	R3D	UCF101	None	0.35	0.39	0.39
KD	C3D	UCF101	R3D	0.55	0.56	0.56
KD	C3D	UCF101	R(2+1)D	0.54	0.54	0.54
Scratch	C3D	UCF101	None	0.48	0.53	0.54
KD	R(2+1)D	UCF101	C3D	0.43	0.45	0.45
KD	R(2+1)D	UCF101	R3D	0.41	0.44	0.45
Scratch	R(2+1)D	UCF101	None	0.41	0.44	0.44

## Data Availability

No new data were created or analyzed in this study. Data sharing is not applicable to this article.
